# Nutritional Status in a Group of Patients with Wounds Due to Diabetic Foot Disease and Chronic Venous Insufficiency

**DOI:** 10.3390/jcm14010043

**Published:** 2024-12-25

**Authors:** Mateusz Skórka, Dariusz Bazaliński, Paweł Więch, Stanisław Kłęk, Dorota Kozieł, Regina Sierżantowicz

**Affiliations:** 1 St. Luke’s Regional Hospital, Independent Community Health Care Centre, 33-100 Tarnów, Poland; skorka.mateusz@op.pl; 2 Institute of Health Sciences, College of Medical Sciences, University of Rzeszów, 35-310 Rzeszów, Poland; dbazalinski@ur.edu.pl; 3 Podkarpackie Specialist Oncology Centre, Specialist Hospital in Brzozów, 36-200 Brzozów, Poland; 4 Clinic of Oncological Surgery, Maria Skłodowska-Curie National Research Institute of Oncology—Kraków Branch, 31-034 Kraków, Poland; klek@poczta.onet.pl; 5 Collegium Medicum, Jan Kochanowski University, 25-369 Kielce, Poland; dorota.koziel@wp.pl; 6 Department of Surgical Nursing, Medical University of Białystok, 15-274 Białystok, Poland; renatasierz@wp.pl

**Keywords:** chronic wounds, nutritional status, nursing

## Abstract

**Background:** Wound healing is a complex physiological process that begins immediately upon injury. Nutritional status significantly affects the course of regenerative processes. Malnutrition can prolong the inflammatory phase, limit collagen synthesis, and increase the risk of new wound formation. The issue of malnutrition is becoming increasingly prevalent and remains a significant concern, particularly among older adults dealing with chronic conditions. **Methods**: The study was conducted at the Wound Treatment Clinic of the Specialist Hospital at the Podkarpackie Oncology Center in Brzozów, Poland, over 12 months (31 December 2022 to 31 December 2023). A prospective assessment was carried out on 106 patients with chronic wounds. The sample selection was purposeful, based on the following criteria: individuals with hard-to-heal vascular wounds related to diabetic foot disease or venous insufficiency, who provided informed consent to participate after reviewing the study concept. The assessment included a questionnaire and biochemical blood analysis. Further evaluations covered wound characteristics and classification based on clinical scales. The morphotic and biochemical blood parameter assessment included albumin concentration, hemoglobin, C-reactive protein (CRP), and the nutritional risk index (NRI). **Results**: A larger wound area was associated with lower morphotic values in both groups. Exudate levels and severity in chronic venous insufficiency (CVI) patients and diabetic foot disease (DFD) were associated with lower hemoglobin, albumin, and NRI values. At the same time, the depth of tissue structure damage correlated with the measured biochemical parameters. **Conclusions**: NRI values and morphotic blood parameters, along with albumin, hemoglobin, and CRP levels, are closely associated with wound characteristics, including surface area, exudate level, and the severity of tissue destruction. The greater the destruction of tissue structures, the higher the risk of malnutrition and wound infection, as indicated by biochemical assessment.

## 1. Introduction

Balanced nutrition and dietary fortification have an undeniable impact on the stages of wound healing. Poor nutritional status disrupts each of these phases, leading to a prolonged inflammatory phase, limited fibroblast proliferation, and insufficient collagen production, which delays granulation formation. Chronic wounds are undoubtedly a source of pain and reduced quality of life, playing a role in worsening nutritional status causing inability to work and earn a living, as well as social isolation, which may lead to stress and mental disorders and increased healthcare costs borne by national health systems [1,2,3,4]. In Europe and North America, malnutrition affects between 1% and 15% of older adults living at home, but this issue rises to 25–60% among seniors in care facilities, especially during hospitalization [1]. According to Kłęk et al. [5], malnutrition is a well-documented negative prognostic factor in both the general population and various patient groups.

According to the definition proposed by the expert panel of the European Society for Clinical Nutrition and Metabolism (ESPEN), malnutrition is a condition resulting from inadequate absorption or intake of nutrients, predisposing individuals to alterations in body composition, including a reduction in fat-free mass (FFM) and body cell mass (BCM). This, in turn, leads to impairments in both the physical and mental functions of the body, adversely affecting the outcomes of treatment for underlying disease [6].

This condition significantly increases the risk of complications, such as pressure ulcers and infections, and also impacts quality of life. This position has been confirmed by experts focused on malnutrition issues [7,8,9]. In addition, there are other factors that can significantly impact disruptions in the wound healing process [10].

Factors influencing the worsening of wound healing outcomes can be categorized as local or systemic. Local factors include those that directly affect the wound’s characteristics, such as oxygen supply to the wound, blood circulation, and bacterial contamination or infection. Systemic factors encompass components defining an individual’s overall health or disease status that impact their regenerative capacity, including age, gender, stress, biochemical markers (e.g., albumin, C-reactive protein, hemoglobin), chronic diseases (e.g., diabetes, heart failure, renal failure, obesity), medications (e.g., steroids, chemotherapy), addictions (e.g., alcoholism, smoking), conditions impairing immunity (e.g., cancer, radiotherapy), and malnutrition. These factors can significantly hinder the healing process and contribute to categorizing a wound as hard to heal [11,12].

Issues related to malnutrition, intense demographic shifts in populations, and the coexistence of hard-to-heal wounds are deeply interconnected [6].

According to estimates, between 1.5 and 2 million people in Europe suffer from hard-to-heal wounds, while in the United States, this issue affects as many as 3 to 6 million people, leading to significant morbidity and mortality [13,14]. Skin damage and associated wounds most commonly occur on the lower extremities and are usually the result of vascular angiopathies associated with chronic diseases. It is estimated that approximately 57% to 80% of leg ulcers are caused by venous insufficiency, 10% to 25% by arterial atherosclerosis, and 5% to 12% are related to diabetic angiopathy [15]. Gau et al. [16] assessed the impact of nutritional status on the risk of amputation in a group of 478 patients with diabetic foot disease (DFD). In their study, the authors used the Mini-Nutritional Assessment (MNA) scale, among other tools. It was shown that malnutrition significantly increased the risk of amputation, with each one-point decrease on the MNA scale further raising this risk by an additional 23% [17].

Assessment of nutritional status and treatment of potential nutritional deficiencies should be standard procedures in the care of patients with wounds. Available data highlight the significant importance and necessity of implementing recommended methods in this regard [18,19,20,21]. The issue of nutritional status in individuals with chronic wounds remains a current and inexhaustible area of research, especially in terms of monitoring nutritional status and its impact on the healing process and potential local complications. The aim of the study was to assess the nutritional status in patients with concurrent wounds in the course of diabetic foot disease and chronic venous insufficiency using (selected) biochemical markers.

## 2. Materials and Methods

### 2.1. Ethics

This study was approved by the Director of the Specialist Hospital at the Podkarpackie Oncology Center in Brzozów, Poland, and received a positive opinion from the Bioethics Committee at the University of Rzeszów (RESOLUTION no. 2023/03/0013 dated 1 March 2023).

During the study, the guidelines of the Helsinki Declaration were followed. Participants were informed about the purpose of the study and provided informed consent before the study began, with the option to withdraw at any time without providing a reason [22].

### 2.2. Subjects

The study concept involved a prospective observation of patient groups conducted at a purposively selected healthcare facility in the Podkarpackie Voivodeship (Surgical Clinic, Wound Treatment Clinic, Podkarpackie Oncology Center in Brzozów, Poland), which provides outpatient treatment for patients with chronic wounds of various etiologies.

Patients with chronic wounds (who consented to participate in the study after reviewing the study concept) were qualified for the research. These included individuals with venous ulcers (classified according to CEAP classification C6, active ulcer with a wound area ≥ 10 cm²) and patients with diabetic foot disease who met the inclusion criteria for the study (wound area ≥ 5 cm², due to the smaller body area and higher risk of damage to deeper structures). Additional criteria included a minimum wound duration of 6 weeks and the absence of contraindications to participation (systemic infection signs: fever > 38 °C, heart rate > 90/min, leukocytosis > 12,000 or <4000). Also excluded were patients with renal insufficiency (creatinine > 1.6 mg/dL) or heart failure NYHA class III and IV, as well as patients with post-minor amputations of the foot.

The observation and examination of patients with wounds constituted the initial stage of therapeutic activities conducted within the wound treatment clinic. The methodological framework of the study did not include long-term observational research. Instead, the focus was placed solely on the current assessment of the nutritional status of patients with coexisting wounds.

**Figure 1:**
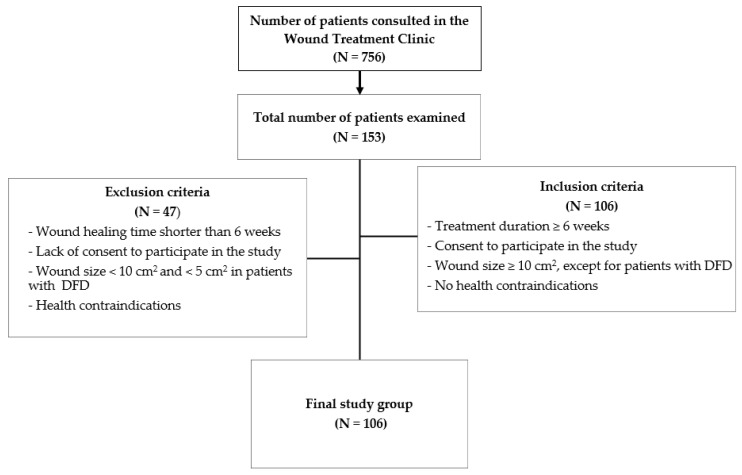
Graphical description of the selection of the study group.

From a group of 756 patients consulted and treated at the wound treatment clinic over a 12-month period (4317 visits), individuals with wounds related to cancer, skin infections, pressure ulcers, and complicated postoperative wounds were excluded. A total of 153 patients with diagnosed chronic wounds were initially selected for the study, from which 47 patients were excluded, mainly due to shorter wound treatment duration, smaller wound sizes, or missing data in the required questionnaires. The final study was conducted on a group of 106 patients, as illustrated in Figure 1 below.

### 2.3. Statistical Analysis

The calculations were performed using IBM SPSS Statistics 22. A statistical significance threshold of *p* < 0.05 was set. The normality of distributions in each group was verified using the Shapiro–Wilk test. The absence of normal distribution for most variables required the use of non-parametric methods.

### 2.4. Data Collection

The prepared scientific research questionnaire consisted of two parts, A and B. Part A included sociodemographic data (age, gender, place of residence, economic status, marital status, and education level). Additionally, it assessed the level of self-care (Barthel Index). The subsequent section evaluated the wound area, exudate level, and depth of tissue damage using scales from the National Pressure Injury Advisory Panel/European Pressure Ulcer Advisory Panel (NPIAP/EPUAP*) and Wagner. Part B contained results from biochemical blood tests, such as blood count, C-reactive protein (CRP), albumin concentration, and the result of the calculated nutritional risk index (NRI). Biochemical tests were performed in a single laboratory, with results presented according to the applicable SI standards.

The Barthel Scale is used to assess a patient’s functional ability and capacity for self-care. It evaluates 10 activities of daily living, also identifying potential deficits, which are associated with specific tasks where the patient may require assistance. The maximum score (85–100) indicates full self-care capacity, while a score of 0–25 points indicates an inability to perform self-care, and a score of 30–80 points indicates limited self-care [23].

The Wagner Classification is a simple six-stage system used to classify foot damage in diabetic foot disease. It is based on the assessment of wound depth and the extent of necrotic tissue, encompassing six stages of progression: Stage 0, high-risk foot; Stage 1, superficial ulcer; Stage 2, ulcer with associated inflammation of the skin and subcutaneous tissues; Stage 3, as above, with additional bone inflammation or foot abscess; Stage 4, limited dry or wet necrosis; and Stage 5, extensive necrosis, indicating the need for amputation [24].

Hemoglobin is a protein that has the ability to bind, transport, and deliver oxygen essential for the body’s function. Reference values for hemoglobin are as follows: women,12.0–16.0 g/dL; and men, 13.0–18.0 g/dL. The degree of anemia is assessed based on hemoglobin level [25].

CRP is C-reactive protein, synthesized in the liver in response to pro-inflammatory cytokines and released into the blood during the early stages of an inflammatory reaction. It is a sensitive marker that allows monitoring of the evolution of inflammation and rapidly normalizes when the inflammatory response subsides. The reference value for CRP is 0.00–5.00 mg/L [26].

Albumin is a protein that dominates in plasma, constituting 60% of plasma proteins. Its concentration in serum below 3.5 g/dL is the most commonly cited indicator of malnutrition. Hypoalbuminemia should not be considered solely as a consequence of malnutrition but as an indicator of the severity of illness and the body’s hydration status. Reference value: 3.5–5.2 g/dL [27]. Reference values are considered indicative of normal nutritional status globally.

The nutritional risk index (NRI) is a recommended indicator for assessing the nutritional status and risk of individuals at risk of malnutrition. Using data on albumin levels and current body weight, the risk of malnutrition can be calculated using the following formula:

NRI = (1.519 × serum albumin concentration [g/L]) + (41.7 × current body weight) [28].

The result allows for the assessment of nutritional status:

- NRI > 100: normal nutritional status,
- NRI = 97.5–100: mild malnutrition,
- NRI > 83.5–97.5: moderate malnutrition,
- NRI < 83.5: severe malnutrition.

Conditionally used to assess wounds on the lower legs, considering tissue destruction and the absence of a preferred assessment tool (1°, superficial wound; 2°, partial-thickness skin wound; 3°, full-thickness skin wound; and 4°, wound with exposed tendon or bone).

## 3. Results

### 3.1. Characteristics of the Respondents

**Figure 2:**
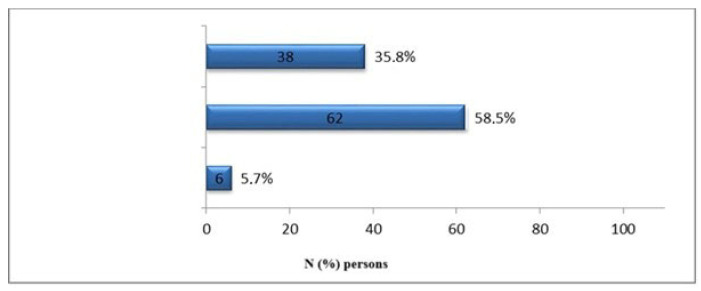
Assessment of participants according to the Barthel scale.

For statistical analysis, 106 participants were included, of which 57.5% (N = 61) were men and 42.5% (N = 45) were women, with an average body mass index (BMI) of 32.29 ± 7.55 kg/m². The participants’ ages varied between 35 and 93 years, with a mean age of 67.03 ± 11.43 years. Among the patients with chronic wounds, 75.5% (N = 80) were urban residents, while 24.5% (N = 26) lived in rural areas. Vocational education was reported by 37.7% (N = 40) of participants with chronic wounds. Basic education was reported by 30.2% (N = 32), secondary education by 26.4% (N = 28), and higher education by 5.7% (N = 6). Economic status was varied: 87.7% (N = 93) had an income below the national average, 11.3% (N = 12) had an income at the national average, and 0.9% (N = 1) had an income above the national average. Self-care capacity, as assessed by the Barthel scale, averaged 75.44 ± 22.92 points, with scores ranging from 5 to 100. According to the established norms, 58.5% (N = 62) of patients were found to have a self-care deficit, 35.8% (N = 38) were unable to self-care, and 5.7% (N = 6) had full self-care capacity, as shown in Figure 2.

### 3.2. Wound Characteristics

The presented study group was treated for wounds associated with chronic venous insufficiency (CVI) and diabetic foot disease (DFD). The CVI group comprised 52.8% (N = 56) of the participants, while the DFD group consisted of 47.2% (N = 50). The average wound area was 55.65 ± 87.96 cm², with a minimum of 5 cm² and a maximum of 625 cm². Based on the NPIAP/EPUAP classification, the participants had an average score of 2.45 ± 0.62 points.

The wounds of the studied patients were located on the foot in 48.2% (N = 50) (DFD), on the lower leg in 42.5% (N = 45), and around the ankles in 9.3% (N = 11) (CVI), as seen in Table 1.

In patients with chronic venous insufficiency, the average wound area was higher, measuring 89.61 cm² (*p* < 0.0001), and the wounds were shallower, primarily involving full-thickness skin damage. Moderate to high levels of exudate were observed in 59.4% (N = 63) of the participants, more frequently in those with chronic venous insufficiency, accounting for 48.2% (N = 27) and 23.2% (N = 13) of the patients. Differences between wound type and exudate levels were significant (*p* = 0.0167), as shown in Table 2. Analyzing exudate levels on a quantitative scale (0–4 points), they were found to be higher (2.14 points) in the CVI group than in the DFD group (1.56 points), with a difference of *p* = 0.0009.

In patients with diabetic foot disease, the average wound area was 17.62 cm² (Table 3). A small amount of exudate was observed in 29.2% (N = 31) of all patients, with a higher frequency (40.0%, N = 20) in the diabetic foot disease group. Significant differences were found between the tissue damage assessment scale (NPIAP/EPUAP) and wound type (*p* = 0.0010). Deeper tissue damage was more common in patients with diabetic foot disease (2.68 points) compared to those with chronic venous insufficiency (2.24 points), as can be seen in Table 4. According to the Wagner classification, superficial ulcers (Wagner 2) were confirmed in 40.0% (N = 20) of the patients, while deep penetrating ulcers involving tendons, bones, or joints (Wagner 3) were observed in 38.0% (N = 19). A small number of patients with diabetic foot disease had deep ulcers with abscesses or osteomyelitis (Wagner 4) 22.0% (N = 11).

### 3.3. Laboratory Tests

**Figure 3:**
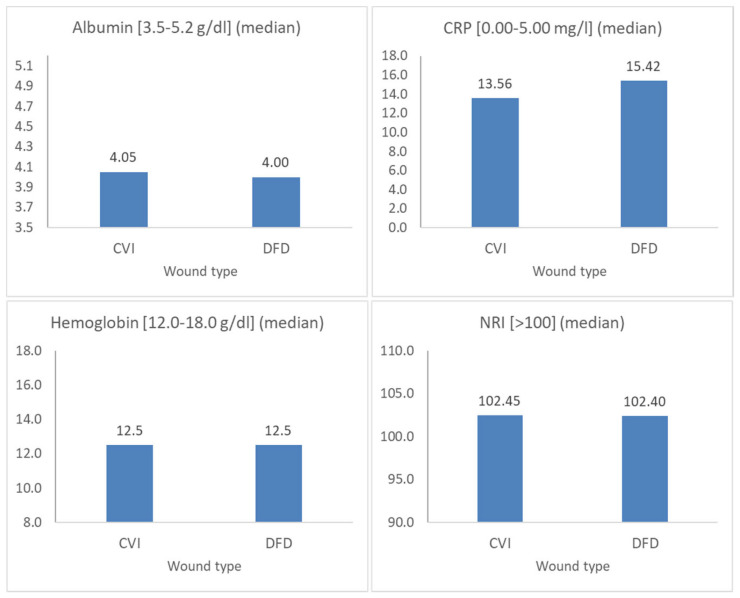
Comparison of biochemical parameters between the patients with CVI and those with DFD (median values).

It was shown that there were no differences in the biochemical parameters between the patients with CVI and those with DFD, particularly in measurements of albumin concentration, hemoglobin, CRP, or NRI. The corresponding test results (Mann–Whitney U test values and *p*) are as follows: albumin U = 1307.0, *p* = 0.556; HGB U = 1358.5, *p* = 0.793; CRP U = 1291.0, *p* = 0.490; NRI U = 1294.5, *p* = 0.504 (Table 5). The data are shown in Figure 3.

### 3.4. Wound Components and Their Correlation with the Applied Scales and Parameters

In patients with chronic venous insufficiency (CVI), a larger wound area was significantly associated with lower levels of albumin (*p* = 0.0126), hemoglobin (*p* = 0.0095), and NRI (*p* = 0.0115). For patients with diabetic foot disease (DFD), an increase in wound area was similarly linked to significantly lower blood morphotic values, including albumin (*p* < 0.0001), hemoglobin (*p* < 0.0001), and NRI (*p* < 0.0001) (Table 6). CVI patients with higher levels of exudate had significantly lower hemoglobin levels (*p* = 0.0186), while DFD patients with higher levels of exudate had substantially lower values of albumin (*p* = 0.0040), hemoglobin (*p* = 0.0285), and NRI (*p* = 0.0018) (Table 7).

**Figure 4:**
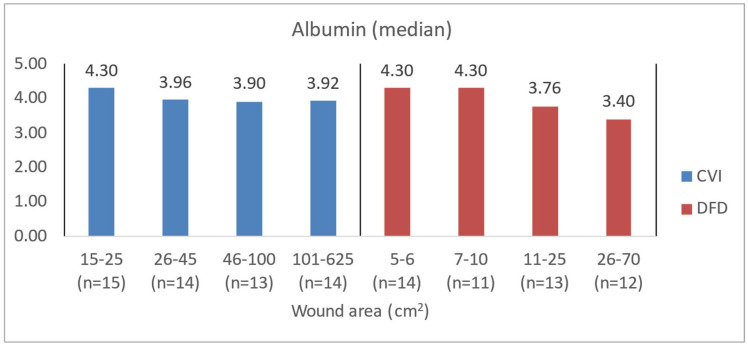
Median albumin values in CVI and DFD patient groups with different wound areas.

**Figure 5:**
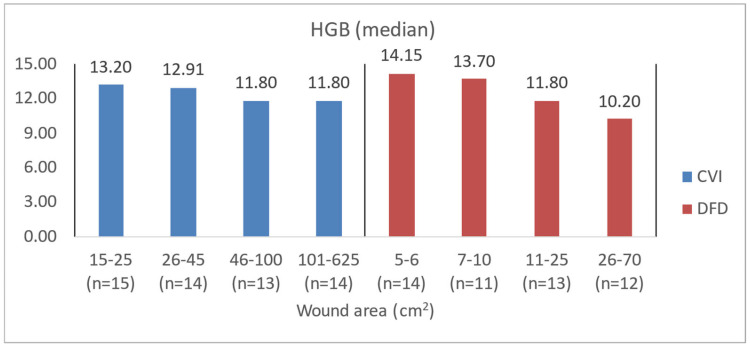
Median HGB values in CVI and DFD patient groups with different wound areas.

**Figure 6:**
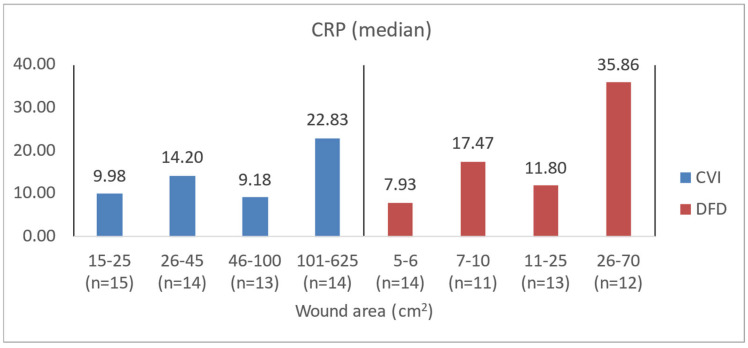
Median CRP values in CVI and DFD patient groups with different wound areas.

**Figure 7:**
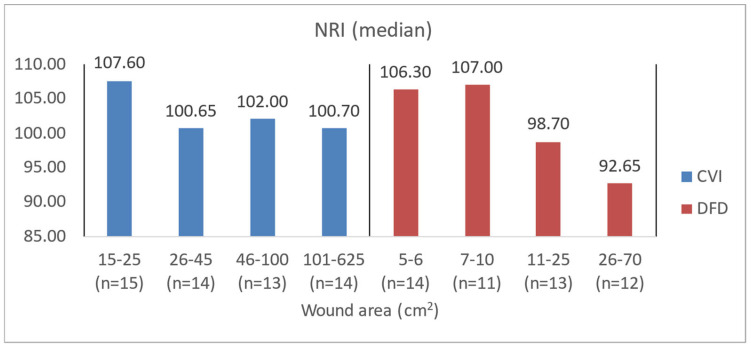
Median NRI values in CVI and DFD patient groups with different wound areas.

To illustrate the difference in blood count levels across area groups, the figures below show the median parameter values. Groups were created by dividing patients with CVI and DFD into four approximately equal categories. Similar charts were created to illustrate differences in wound area or NPIAP/EPUAP scale levels (Figure 4, Figure 5, Figure 6 and Figure 7).

Increased exudate within the wound is most often a result of inflammation and/or edema. Venous ulcers usually determine edema. Exudate and potential inflammation may cause disturbances associated with protein loss, leading to anemia. The obtained data indicate that, in the DFD group, morphotic parameters were impaired, resulting in changes in nutritional status (*p* < 0.05).

**Figure 8:**
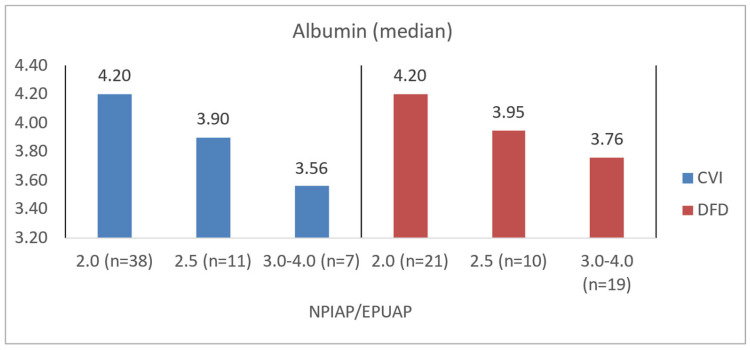
Median albumin values in CVI and DFD patient groups with different NPIAP/EPUAP values.

**Figure 9:**
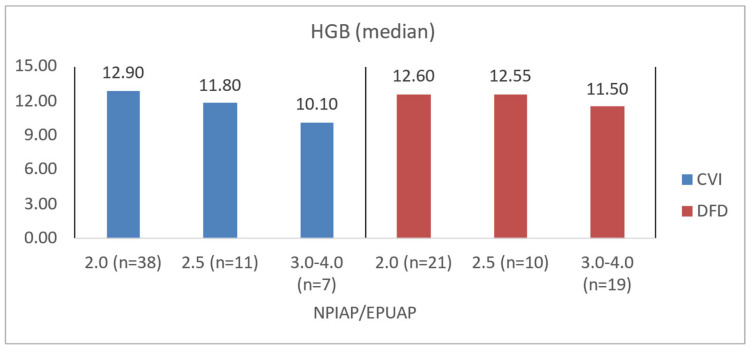
Median HGB values in CVI and DFD patient groups with different NPIAP/EPUAP values.

**Figure 10:**
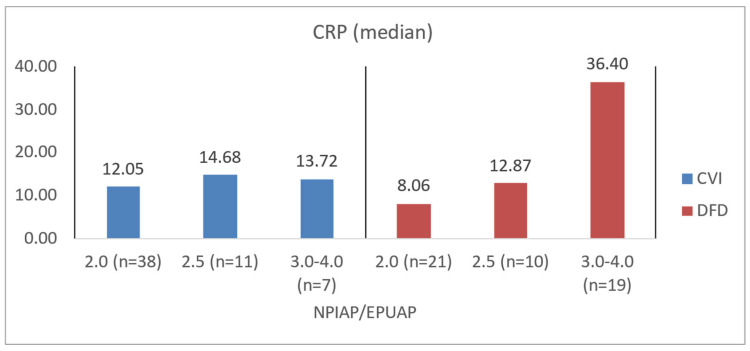
Median CRP values in CVI and DFD patient groups with different NPIAP/EPUAP values.

**Figure 11:**
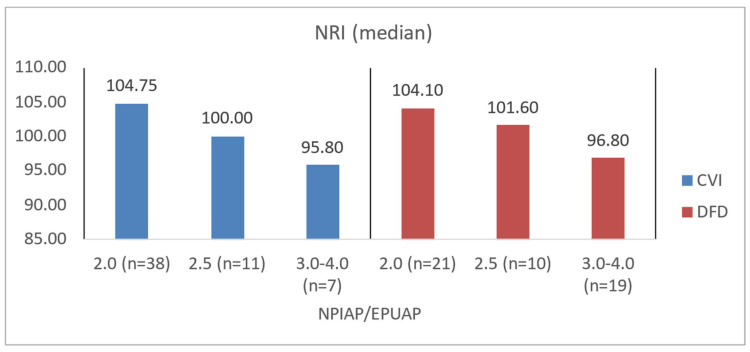
Median NRI values in CVI and DFD patient groups with different NPIAP/EPUAP values.

Among CVI patients, those with higher NPIAP/EPUAP scores also demonstrated significantly lower levels of albumin (*p* = 0.0077), hemoglobin (*p* = 0.0123), and NRI (*p* = 0.0087). DFD patients with greater tissue destruction per the Wagner scale similarly showed lower albumin levels (*p* = 0.0233), a lower NRI (*p* = 0.0107), and significantly higher CRP levels (*p* = 0.0021) (Table 8). In the assessment of tissue damage depth and biochemical morphotic parameters, it was shown that in the DFD group, there was a higher risk of disturbances related to nutritional status and potentially a higher risk of wound infection (*p* = 0.0021) compared to the CVI group (*p* = 0.7366). The obtained data are also presented for better illustration in Figure 8, Figure 9, Figure 10 and Figure 11.

## 4. Discussion

Malnutrition, the intensity of aging processes, and the coexistence of chronic wounds are closely interconnected, suggesting a potential link in this area. It has been shown that dietary fortification, including protein intake, is essential for maintaining proper platelet function, fibroblast proliferation, and wound remodeling. Protein-calorie deficiencies can impair the formation of capillaries and collagen synthesis, ultimately delaying regenerative processes and prolonging the inflammatory phase [13,29].

Malnutrition is often underestimated as an issue among both younger and older patients. To better objectively and comprehensively assess patients’ nutritional status, various recommended scales, tools, and biochemical blood parameters are used, which are compared and discussed in more detail in the following sections. This information offers an opportunity to better understand their role and significance in evaluating patient health.

In this study, nutritional status was assessed using biochemical parameters in a group of individuals with chronic vascular-origin wounds, showing no significant differences between patients with CVI and DFD in albumin concentration (*p* = 0.5556), hemoglobin levels (*p* = 0.7928), C-reactive protein (CRP) levels (*p* = 0.4903), or the nutritional risk index (NRI) (*p* = 0.5041). A detailed analysis of the results highlights that, while albumin levels were within normal ranges for most patients, half of the participants had hemoglobin levels below the norm, and the CRP level was elevated in the majority. The nutritional risk index (NRI) indicated a normal status in 60 and 60.7% of cases, with approximately 40% showing varying degrees of malnutrition, categorized as mild, moderate, or severe. Mild malnutrition was more commonly observed in the CVI group, with 11 patients, whereas in the DFD group, 5 patients were affected. Moderate malnutrition was observed in 10 patients from the CVI group and 12 from the DFD group. Severe malnutrition was found in one patient from the CVI group and in as many as three patients from the DFD group.

The issue of hemoglobin concentration deserves attention. Anemia is defined as a hemoglobin (HGB) level below 13 g/dL in men, below 12 g/dL in women, or below 12 g/dL for individuals over 65, regardless of sex [30]. The hemoglobin levels observed in the patient groups in this study were only marginally above the accepted norm, with a mean of (12.44 ± 1.71 g/dL). Studies by other authors on larger sample sizes could offer valuable insight into this discussion. Research by Gezawa et al. [31] on 336 Nigerian patients with diabetes hospitalized for DFD assessed anemia prevalence and its impact on clinical outcomes. Conducted in six high-level healthcare facilities, their findings confirmed anemia in more than half of the DFD cohort. The study showed a significant association between anemia and impaired wound healing (*p* < 0.009), amputation (*p* < 0.036), and mortality risk (*p* < 0.034) [32,33]. The authors also highlighted the need for future studies to focus on the effects of anemia correction on improving outcomes in hospitalized DFD patients. Our study did not focus on evaluating anemia’s consequences or tracking the outcomes of patients with chronic wounds. However, referencing findings from other authors suggests that this parameter warrants close attention. Although anemia values may fall within the lower normal range, prolonged anemia could lead to adverse health outcomes. Monitoring and appropriate therapeutic interventions in such cases could be crucial for improving the patients’ overall condition and wound healing process.

In both groups, it was found that the surface area and depth of tissue destruction predispose individuals to lower levels of albumin, hemoglobin, and NRI. These findings are consistent with studies by other authors, such as Singh et al. [31] and Palmieri et al. [34], who emphasize the validity of targeted nutritional supplementation and its impact on reducing wound size in malnourished patients. They also highlight the significant relationship between nutritional status and the regenerative potential of the resulting injury [13,35,36]. In the context of the patients studied, the relationship between nutritional status and exudate levels was also an important issue addressed in our research, as it significantly impacts wound healing. The study demonstrated an increase in inflammatory markers associated with the wound area and tissue destruction levels. Diabetic foot disease results from diabetes complications, with tissue damage progressing at a rapid pace, leading to a localized inflammatory response induced by tissue infection, often caused by microorganisms of cutaneous origin. Chronic inflammation is associated with the release of acute-phase proteins, which may negatively correlate with low albumin levels and, consequently, the development of anemia. In the DFD group, anemia may also be linked to other variables, including multimorbidity.

Exudate mainly consists of water but also contains other macro- and micronutrients, such as platelets, plasma proteins, glucose, growth factors, and metabolic waste products. The intensive production of exudate, particularly in the case of large wounds, is mainly associated with wound infection, leading to an increase in C-reactive protein (CRP) levels. These findings are supported by both national and international literature, emphasizing the need for an integrated approach to monitor nutritional status and manage exudate in the wound healing process [37,38]. Exudate in the wound correlates with infection and vascular insufficiency, which predispose to limb edema. Increased exudate is a clinical manifestation of infection in the wound. Mutailipu et al. [39], in a cross-sectional study of 577 patients with DFD, analyzed their nutritional status. The patients were divided into three groups based on the Wagner classification: Wagner I–II (19.06% of the total), Wagner III (38.13%), and Wagner IV–V (42.8%). Comparisons between the groups revealed that hemoglobin, albumin, prealbumin, HbA1c, and leukocytes correlated with the Wagner classification (*p* < 0.001), with patients in the Wagner IV–V group having worse nutritional status than those in Wagner I–III. In our study, most patients in the DFD group were classified as Wagner II (40.0%) and Wagner III (38.0%). A portion of the DFD group presented with deep ulcers with abscesses or osteomyelitis (Wagner IV) (22.0%). It was confirmed that the level of tissue destruction, based on the NPIAP/EPUAP and Wagner scales, was closely associated with albumin, hemoglobin, NRI, and CRP levels, which has also been confirmed by other authors [32,40,41]. Reviewing the literature, one might question the concept that albumin concentration, as a marker of nutritional status, is not sensitive enough to provide an accurate and definitive diagnosis. C-reactive protein (CRP) is primarily produced by hepatocytes during the acute phase of inflammation in response to cytokine stimulation (IL-1, IL-6, and TNF-α). Following a conformational change mediated by activated platelets, CRP becomes a pro-inflammatory protein capable of recruiting additional inflammatory cells. Its half-life is approximately 19 h, and its blood levels rise within 6 h of the onset of inflammation or tissue damage. Within 2–3 days of the inflammatory response, CRP levels increase rapidly. The assessment of serum albumin concentration is often used as a marker of disease presence, persistence, or improvement, while CRP levels serve as an indicator of ongoing inflammation [42,43]. Albumin is the most abundant protein in plasma and has a regulatory role in the distribution of body fluids, acid-base physiology, and binding of essential components in the bloodstream. It has a long half-life of 18–21 days, making it unsuitable for monitoring rapid changes in nutritional status in patients undergoing nutritional treatment [44]. Translating these facts to practice, it is important to emphasize that treating patients with chronic wounds is a prolonged and time-consuming process. The possibilities of albumin concentration assessment, its reproducibility, and low cost may still make it a valuable prognostic marker for assessing the nutritional status of patients with wounds. Protein supplementation and its monitoring are particularly important for patients with diabetes.

C-reactive protein (CRP) is produced by hepatocytes and is commonly used to assess inflammation. It has been previously noted that acute-phase concentrations of proteins, such as CRP, tend to rise in inflammatory conditions, while albumin concentrations tend to decline. As indicated by the obtained results, this process requires periodic monitoring of biochemical parameters, which is particularly important in the DFD patient group due to the higher risk of potential complications in the wound healing process. The hypothesis was made that higher levels of inflammatory markers would negatively correlate with albumin levels. It was noted that chronic inflammation associated with high CRP was negatively correlated with albumin concentrations. Similar observations were presented by Sheinenzon et al. [45].

It is essential to recognize that diabetes is currently the most common cause of kidney problems, both in developed and developing countries. In the early stages of diabetes, the kidneys enlarge, and the glomerular filtration rate (GFR) exceeds the expected normal range. End-stage renal disease (ESRD) is a significant factor contributing to ulcers and increases the frequency of amputations by 2.5 to 3 times. Therefore, additional monitoring of parameters such as urea and creatinine is a crucial approach in this patient group [46]. Considering that albumin levels may be altered in advanced kidney failure, liver diseases, and cancer-related conditions, individuals who presented with disorders related to the urinary system and liver failure in the initial assessment were excluded from the study.

From our observations, it appears that elderly patients do not always show visible signs of malnutrition, which may lead to the early signs of this condition being overlooked during routine checks by medical staff. Special consideration should be given to identifying nutritional problems in individuals with significantly limited self-care, obese individuals, and those with physical or cognitive limitations [47].

Similar observations are presented by other authors, who emphasize the cascade of negative effects, such as impaired muscle function, delayed wound healing, and reduced quality of life in this group of patients. Therefore, paying special attention to the nutritional status of older individuals is crucial in preventing further health complications and improving the effectiveness of ongoing treatment [48,49].

In summary, nutritional status and manifestations of potential malnutrition can vary across different patient groups. In our own research, it was shown that patients with pressure injuries are most at risk of experiencing this issue [10,50]. Considering the future demographic changes and projections regarding older individuals and comorbidities, the number of patients with DFD and CVI will continue to rise, and these individuals will constitute an increasingly larger portion of society. In light of this fact, an important aspect of the conducted studies was to raise awareness about the nutritional status in these patient groups. It is crucial that, in addition to targeted treatments for wound care, healthcare personnel’s clinical vigilance regarding potential malnutrition should always be considered, especially when the wound, its surface area, depth of tissue destruction, or exudate level are advanced, even when mobility and the ability to consume food are preserved in the patient.

### Limitations

The results of the present study have certain limitations, which stem from both the adopted research concept and the available budget. Expanding the study to include additional centers and conducting long-term observations of patients’ nutritional status during treatment could offer an interesting option and added value for future scientific research. Such an approach would enable a more comprehensive understanding of the dynamics of changing nutritional status and its impact on the wound healing process, as well as allow for the identification of potential risk factors and the development of more effective management strategies.

## 5. Conclusions

The surface area and degree of tissue damage in the wound bed correlate with nutritional status parameters. In both groups, a larger wound area was associated with lower levels of albumin, hemoglobin, and the NRI index. Increased exudate levels in the wound were linked to lower values of hemoglobin, albumin, and the NRI index. The level of tissue destruction negatively impacted the nutritional status parameters, leading to lower values in NRI and biochemistry, as well as higher levels of acute-phase proteins (CRP), indicating the risk of malnutrition and the presence of inflammation.

## Figures and Tables

**Table 1:** Wound location and type (N = 106).

	Type of Wound					
	Chronic Venous Insufficiency (CVI)		Diabetic Foot Disease (DFD)		Total	
Location of the wound	N	%	N	%	N	%
Foot	0	0.0%	50	100.0%	50	48.2%
Lower limb (ankle)	11	19.6%	0	0.0%	11	9.3%
Shin	45	80.4%	0	0.0%	45	42.5%
Total	56	100.0%	50	100.0%	106	100.0%
	F = 130.104; *p*(F) < 0.0001					

CVI, chronic venous insufficiency; DFD, diabetic foot disease; N, number of individuals; F, Fisher-Freeman-Halton test value; *p*, statistical significance level.

**Table 2:** Exudate level and wound type (N = 106).

			Type of Wound		Overall
			Chronic Venous Insufficiency (CVI)	Diabetic Foot Disease (DFD)	
Exudate level	None	N	1	5	6
		%	1.8%	10.0%	5.7%
	Minimal	N	11	20	31
		%	19.6%	40.0%	29.2%
	Moderate	N	27	19	46
		%	48.2%	38.0%	43.4%
	Large	N	13	4	17
		%	23.2%	8.0%	16.0%
	Very large	N	4	2	6
		%	7.1%	4.0%	5.7%
Overall		N	56	50	106
		%	100.0%	100.0%	100.0%
F = 11.513; *p*(F) = 0.0167					

CVI, chronic venous insufficiency; DFD, diabetic foot disease; N, number of individuals; F, Fisher-Freeman-Halton test value; *p*, statistical significance level.

**Table 3:** Surface area vs. wound type (N = 106).

		Wound Type								*p*
		N	M	SD	Me	Min.	Max.	Q1	Q3	
Wound area [cm²]	CVI	56	89.61	110.00	45.00	15.00	625.00	25.00	102.50	Z = −6.408; *p* < 0.0001
	DFD	50	17.62	14.30	11.00	5.00	70.00	6.00	25.00	

CVI, chronic venous insufficiency; DFD, diabetic foot disease; N, number of individuals; M, mean; SD, standard deviation; Me, median; Min, minimum value; Max, maximum value; Q1, first quartile; Q3, third quartile; Z, Mann–Whitney test value; *p*, statistical significance level.

**Table 4:** Classification according to NPIAP/EPUAP and wound type (N = 106).

		Wound Type								*p*
		N	M	SD	Me	Min.	Max.	Q1	Q3	
NPIAP/ EPUAP	CVI	56	2.24	0.42	2.00	2.00	4.00	2.00	2.50	Z = −3.285; *p* = 0.0010
	DFD	50	2.68	0.73	2.50	2.00	4.00	2.00	3.50	

CVI, chronic venous insufficiency; DFD, diabetic foot disease; N, number of individuals; M, mean; SD, standard deviation; Me, median; Min, minimum value; Max, maximum value; Q1, first quartile; Q3, third quartile; Z, Mann–Whitney test value; *p*, statistical significance level.

**Table 5:** Biochemical parameters and wound type (N = 106).

		Wound Type								*p*
		N	M	SD	Me	Min.	Max.	Q1	Q3	
Albumin [3.5–5.2 g/dL]	CVI	56	3.99	0.49	4.05	1.78	4.70	3.80	4.35	Z = −0.589; *p* = 0.5556
	DFD	50	3.93	0.53	4.00	2.50	4.80	3.71	4.30	
Hemoglobin (HGB) [12.0–18.0 g/dL]	CVI	56	12.42	1.41	12.50	9.50	15.30	11.55	13.50	Z = −0.263; *p* = 0.7928
	DFD	50	12.47	2.00	12.50	8.30	16.90	11.20	14.20	
CRP [0.00–5.00 mg/L]	CVI	56	23.44	29.11	13.56	0.24	154.57	4.40	33.90	Z = −0.690; *p* = 0.4903
	DFD	50	28.45	32.32	15.42	0.63	130.00	4.58	46.00	
Malnutrition Risk Index (NRI) [>100]	CVI	56	101.76	7.68	102.45	66.80	113.00	99.35	107.30	Z = −0.668; *p* = 0.5041
	DFD	50	100.66	8.24	102.40	78.40	113.80	96.80	107.00	

CVI, chronic venous insufficiency; DFD, diabetic foot disease; N, number of individuals; M, mean; SD, standard deviation; Me, median; Min, minimum value; Max, maximum value; Q1, first quartile; Q3, third quartile; Z, Mann–Whitney test value; *p*, statistical significance level.

**Table 6:** Biochemical parameters vs. wound surface area (N = 106).

Wound Area [cm^2^]	CVI		DFD	
	R	*p*	R	*p*
Albumin [3.5–5.2 g/dL]	−0.331	0.0126	−0.674	<0.0001
Hemoglobin (HGB) [12.0–18.0 g/dL]	−0.344	0.0095	−0.664	<0.0001
CRP [0.00–5.00 mg/L]	0.189	0.1640	0.265	0.0632
Nutritional Risk Index (NRI) [>100]	−0.335	0.0115	−0.647	<0.0001

CVI, chronic venous insufficiency; DFD, diabetic foot disease; R, Spearman’s rank correlation coefficient; *p*, statistical significance level.

**Table 7:** Biochemical parameters vs. exudate level (N = 106).

Exudate Level	CVI		DFD	
	R	*p*	R	*p*
Albumin [3.5–5.2 g/dL]	−0.196	0.1472	−0.400	0.0040
Hemoglobin (HGB) [12.0–18.0 g/dL]	−0.314	0.0186	−0.310	0.0285
CRP [0.00–5.00 mg/L]	0.187	0.1673	0.251	0.0788
Nutritional Risk Index (NRI) [>100]	−0.193	0.1540	−0.431	0.0018

CVI, chronic venous insufficiency; DFD, diabetic foot disease; R, Spearman’s rank correlation coefficient; *p*, statistical significance level.

**Table 8:** Biochemical parameters and NPIAP/EPUAP and Wagner scale (N = 106).

NPIAP/ EPUAP and Wagner Scale	CVI		DFD	
	R	*p*	R	*p*
Albumin [3.5–5.2 g/dL]	−0.353	0.0077	−0.320	0.0233
Hemoglobin (HGB) [12.0–18.0 g/dL]	−0.332	0.0123	−0.167	0.2454
CRP [0.00–5.00 mg/L]	0.046	0.7366	0.425	0.0021
Nutritional Risk Index (NRI) [>100]	−0.347	0.0087	−0.358	0.0107

CVI, chronic venous insufficiency; DFD, diabetic foot disease; R, Spearman’s rank correlation coefficient; *p*, statistical significance level.

## Data Availability

The data presented in this study are available on reasonable request from the corresponding author: skorka.mateusz@op.pl.
